# Phylostratigraphic profiles reveal a deep evolutionary history of the vertebrate head sensory systems

**DOI:** 10.1186/1742-9994-10-18

**Published:** 2013-04-12

**Authors:** Martin Sebastijan Šestak, Vedran Božičević, Robert Bakarić, Vedran Dunjko, Tomislav Domazet-Lošo

**Affiliations:** 1 Laboratory of Evolutionary Genetics, Ruđer Bošković Institute, Bijenička cesta 54, Zagreb, Croatia; 2 Evolutionsbiologie, Biozentrum Abteilung II, Ludwig-Maximilians-Universität München, Großhaderner Straße 2, 82152 Planegg-Martinsried, Germany; 3 Max-Planck-Institut für Evolutionsbiologie, August-Thienemannstrasse 2, 24306 Plön, Germany; 4 School of Informatics, University of Edinburgh, 10 Crichton Street, EH8 9AB Edinburgh, UK

**Keywords:** Genomic phylostratigraphy, Macroevolution, Sensory systems, Vertebrates, Placodes, Neural crest, Zebrafish

## Abstract

**Background:**

The vertebrate head is a highly derived trait with a heavy concentration of sophisticated sensory organs that allow complex behaviour in this lineage. The head sensory structures arise during vertebrate development from cranial placodes and the neural crest. It is generally thought that derivatives of these ectodermal embryonic tissues played a central role in the evolutionary transition at the onset of vertebrates. Despite the obvious importance of head sensory organs for vertebrate biology, their evolutionary history is still uncertain.

**Results:**

To give a fresh perspective on the adaptive history of the vertebrate head sensory organs, we applied genomic phylostratigraphy to large-scale *in situ* expression data of the developing zebrafish *Danio rerio*. Contrary to traditional predictions, we found that dominant adaptive signals in the analyzed sensory structures largely precede the evolutionary advent of vertebrates. The leading adaptive signals at the bilaterian-chordate transition suggested that the visual system was the first sensory structure to evolve. The olfactory, vestibuloauditory, and lateral line sensory organs displayed a strong link with the urochordate-vertebrate ancestor. The only structures that qualified as genuine vertebrate innovations were the neural crest derivatives, trigeminal ganglion and adenohypophysis. We also found evidence that the cranial placodes evolved before the neural crest despite their proposed embryological relatedness.

**Conclusions:**

Taken together, our findings reveal pre-vertebrate roots and a stepwise adaptive history of the vertebrate sensory systems. This study also underscores that large genomic and expression datasets are rich sources of macroevolutionary information that can be recovered by phylostratigraphic mining.

## Background

Sensing environmental stimuli is a pervasive property of cellular organisms. However, complex sensory organs that receive various types of sensory information are predominantly found in animals. Many examples show that the genes and cell types relevant for the development and function of sensory systems in animals are homologous between diverse animal lineages [1-4]. Yet, at the level of sensory organs parallelism is common. A well-known example is the visual system: while the associated transcription factors and sensory cell types are homologous between various animal phyla, at the organ level this system (i.e., eyes) evolved multiple times independently [1,5]. This interplay of old and new often complicates the understanding of organ system evolution in extant animals [2,4,6]. The evolution of the head sensory systems in vertebrates is a prominent instance of this conundrum.

The vertebrate head is a highly derived trait with a high density of sophisticated sensory organs that allow complex behaviour in this lineage [7-9]. The evolutionary origin of head sensory systems is considered one of the major vertebrate innovations [8]. This is reflected in the ubiquity and diversity of modern and ancient sensory systems among vertebrates [2,10-12]. The evolutionary emergence of vertebrate sensory systems is tightly linked to the evolution of cranial placodes and the neural crest [13,14]. Namely, sensory organs arise during early development from these two embryonic tissues that are located adjacent to each other in the cranial region.

**Figure 1:**
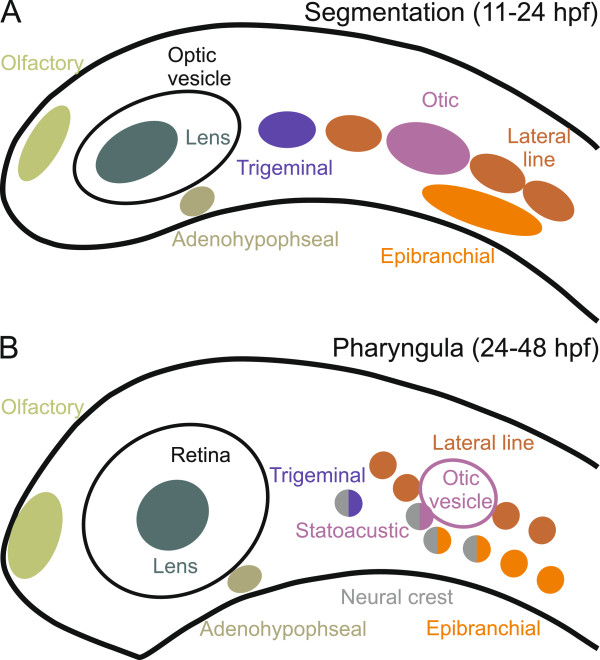
**Cranial placodes and sensory neural crest during head development in zebrafish.** A schematic side view of the zebrafish embryonic head is shown (anterior is to the left and dorsal at the top). Approximate anatomical positions of the individual placodes are reconstructed from the *in situ* hybridizations taken from the ZFIN database [57]. Panel **A** depicts the embryo during segmentation stages (11–24 hours post fertilization). Panel **B** depicts the embryo during the pharyngula stages (24 – 48 hours post fertilization). Distinct placodes are marked by different colors. Ganglia of mixed placodal and sensory neural crest origin are represented by circles where half of the circle is colored in gray (neural crest). The optic vesicle and the retina, which originate directly from the neural tissue, are in black.

During development, the vertebrate neural crest forms at the lateral borders of the neural plate [13,15]. Neural crest cells delaminate from this region, undergo an epithelial-mesenchymal transformation, and migrate to their final destinations, where they give rise to different head structures, such as pigment cells and parts of the cranial and pharyngeal skeleton [13,15]. In addition, the neural crest contributes to the sensory system by providing neurons to the trigeminal and statoacustic ganglia as well as to the proximal ganglia of the facial, glossopharyngeal and vagal nerves [16]. However, the majority of head sensory systems arise from the cranial placodes, which develop anterior to the neural plate and lateral to the neural crest [14]. The placodes develop as mostly paired, ectodermal thickenings that differentiate from a panplacodal primordium [17]. The lens placode gives rise to the lens (vision), olfactory placode to the olfactory system (smell), lateral line placodes to the lateral line system (water flow and pressure in aquatic vertebrates), otic placode to the vestibuloauditory system (balance and hearing), trigeminal placode to part of the trigeminal ganglion (touch, temperature, and sense of pain in the head), epibranchial placodes to the distal ganglia of the facial, glossopharyngeal, and vagal nerves (touch and taste), and adenohypophyseal placode to the adenohypophysis (endocrine function) [6,16-19]. Figure 1 illustrates the anatomic positions of the cranial placodes and the sensory neural crest in the developing head of the zebrafish.

The importance of cranial placodes and neural crest for the evolution of vertebrate sensory systems was originally recognized by Northcutt and Gans [7,9,20]. They proposed that a simultaneous emergence of neural crest and placode-derived tissues in the vertebrate ancestor was one of the key events leading to the origin of vertebrate sensory systems and the vertebrate body plan in general. According to their “New Head Hypothesis”, this sudden jump in complexity from the simple cephalochordate-like ancestor was linked with the ecological shift from a semi-sessile, filter-feeding lifestyle to an active predatory one [7,9,20]. In contrast to the idea of such an abrupt emergence of vertebrate innovations, some researchers recognized that the transition from the chordate to the vertebrate ancestor could have proceeded in a more gradual way [21]. For instance, the presence of a visual system in the chordate ancestor would provide distinct adaptive advantages to the early chordates even without the other neural crest and placode derivatives [21-24]. This line of thinking is summed up in the “Serial Transformation Hypothesis” which assumes that the vertebrate sensory system had a stepwise evolutionary origin, building on the eyes that were acquired before the origin of placodes and neural crest tissues [21,22]. In fact, paleontological records of the Lower Cambrian (515–520 Mya) reveal some pre-vertebrate fossils that possess paired eyes but lack ears and most other placodal and neural crest derivatives [23,24]. However, taxonomic uncertainty, unknown degradation status, and the bias in the preservation of the vertebrate and chordate characters in these fossils preclude reliable conclusions about the succession of events during the chordate-vertebrate transition [25-27].

Similarly to the paleontological record, comparative molecular studies are ambiguous. The regulatory network governing placode development in vertebrates has been well studied [17,19]. It is largely conserved in vertebrates and some of its parts were also found in tunicates [28], while the situation in cephalochordates is less clear [29]. In tunicates, siphon primordia are proposed to be homologous to some cranial placodes (olfactory, otic, and adenohypophyseal) on the basis of expression of homologous genes in these territories [28]. Additionally, some researchers proposed homology between the coronal organ, a sensory organ situated in the oral siphon of tunicates, and vertebrate sensory hair cells, which are derivatives of otic and lateral line placodes [3,30]. Some questions, however, remain open. For instance, the expression patterns of placodal genes could not be easily compared between vertebrates and tunicates [17,18]. Similar to the neural crest many of the placode patterning genes have a conserved sequence and can be found in other bilaterian phyla, where they play a role in sensory system development [14,31]. All of this argues that some elements of the placodal system were present before the origin of vertebrates. However, it is less obvious which parts of the placodal system were present in these groups. It also remains unclear which of the placodes was ancestral [14]. Initially, it was proposed that the ancestral proto-placode was specified as an adenohypophyseal-olfactory placode [18]. However, this contrasts with the developmental finding in the chick embryo that all placodes are initially specified as lens [32].

The neural crest gene regulatory network is conserved among vertebrates, including cyclostomes [15,33]. As one could expect for a conserved regulatory network, some of its parts are present in other chordate groups, i.e*.*, tunicates and cephalochordates [15,34,35]. In addition, some studies report pigmented neural crest-like cells with migratory properties in ascidians [34,36]. These findings led to the hypothesis that the neural crest originated before the dawn of vertebrates [34,36]. On the other hand, homologues of the neural crest genes in tunicates and cephalochordates have different expression patterns in comparison to vertebrates [15]. Moreover, migratory potential alone could not be considered as a definitive marker of the neural crest as this feature is a very ancient process that relies on old gene networks that are conserved among bilaterians [15,37]. It is also notable that the marker used for the detection of the neural crest-like cells in tunicates was not universal and specific for the vertebrate neural crest [15]. Still, a recent study shows that a single regulatory mutation is sufficient to induce the migratory behaviour of the ectodermal cells at the border of the neural plate in *Ciona intestinalis*[38]. Taken together it is clear that the evolutionary origin of the neural crest is still obscure [13].

The evolutionary relationship between cranial placodes and the neural crest is another open question that remains to be solved. Based on developmental similarities, it has been initially proposed that placodes and the neural crest have a common evolutionary origin [7]. For instance, both cell populations have migratory capacity, produce sensory neurons and glial cells, secrete proteoglycans, and emerge at the borders of the neural plate. Alternatively, these similarities could result from a convergent use of transcription factors implying an independent evolutionary origin of the neural crest and cranial placodes [39]. At the moment, neither scenario is supported by sufficient evidence, albeit the case for the independent origin is probably stronger [14,39].

To address the above open questions we performed a phylostratigraphic analysis of the large scale *in situ* hybridization data in zebrafish (*Danio rerio)*. Genomic phylostratigraphy has turned out to be an especially well-suited approach for studying macroevolutionary transitions using genome-scale data [40-45]. This enabled us to simultaneously analyze the adaptive history of all zebrafish sensory organs from the last common ancestor of cellular organisms to the present day zebrafish in unprecedented detail.

## Results

**Figure 2:**
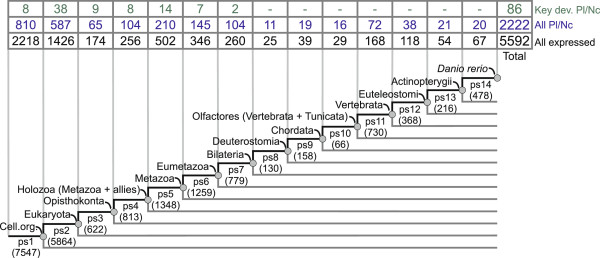
**Phylostratigraphic distribution of the zebrafish genes on the consensus phylogeny.** A consensus phylogeny that spans from the origin of the first cell to *Danio rerio.* Numbers in parentheses denote the total number of genes per phylostrata (ps1-ps14) across the zebrafish genome. The table above the phylogeny shows distributions of different categories of zebrafish genes. The numbers for the total set of genes with spatially restricted expression [57] are shown in the bottom row of the table (black). A subset of genes with expression in the cranial placodes and the neural crest (All Pl/Nc) is shown in the middle row (blue). A set of placode and neural crest key developmental genes (Key dev. Pl/Nc) that are reported in the literature [17,52] is shown in the upper row (green).

We set the stage for the phylostratigraphic analysis by defining a phylogenetic framework of 14 phylogenetic levels (phylostrata) that represent the deuterostomic lineage leading to the zebrafish (Figure 2). This consensus phylogeny is supported by a range of recent studies [46-51] and covers a time span from the origin of the first cell to the terminal lineage, i.e., the genus *Danio* (see *Methods*)*.* Using a bioinformatic pipeline for phylostratigraphic analysis, we performed sequence similarity searches and mapped 20378 zebrafish genes into the corresponding phylostrata (ps) (Figure 2).

To get an initial glimpse of how the essential regulatory genes of the neural crest and placodes are distributed over the phylostratigraphic map, we analyzed the existing knowledge base on key developmental genes in these tissues [17,52] (Figure 2). These genes are commonly used in comparative Evo-Devo approaches, where expression patterns of homologues are compared across lineages [13,14]. This set of 86 genes is not a first choice for the phylostratigraphic analysis due to its relatively small size and inherently biased composition. However, it allowed us to make some important points. As one might expect, protein sequences of these genes turned out to be quite old, predating the diversification of bilaterians (ps7) (Figure 2). A time span from the origin of the eukaryotes up to the origin of metazoans (ps2-ps5) is especially enriched with them (Figure 2, Additional file 1: Figure S1). This is perhaps not surprising given that these phylostrata are known to be replete with transcription factors and patterning genes [42,53]. Evidently, the most interesting span in the phylogeny (ps8-ps14), i.e., the diversification of bilaterians, where one might expect the formation and elaboration of the neural crest and placodes, is completely devoid of genes that are presently known to play an important role in neural crest and placode development (Figure 2). The absence of these genes in younger phylostrata indicates that recovery of the relevant phylostratigraphic signal requires a much broader collection of genes.

It is clear that the total set of genes that shows restricted expression in some developing morphological structure is not constrained to key developmental genes. For instance, large scale whole mount *in situ* hybridizations studies, where genes are more or less randomly tested for gene expressions, uncovered many previously functionally uncharacterized genes with anatomically restricted expression [54]. These genes also tend to have restricted phylogenetic distribution, i.e., they are orphan genes [55,56], and are therefore much more suitable for recovering lineage-specific phylostratigraphic signals [40]. Among vertebrates, currently the best collection of anatomically annotated *in situ* hybridization expression patterns is available for the zebrafish ontogeny [57]. This dataset, recovered from the ZFIN database, contains in total 5592 genes that show regulated expression across zebrafish development (Figure 2). Among these, 2222 genes are expressed in placodes or the neural crest. The distribution of these genes, and the corresponding expression domains that spread over all phylostrata (Figure 2, Additional file 2: Table S1) enabled us to screen the head sensory system tissues for significant phylostratigraphic signals in the full phylogenetic range.

### Pre-vertebrate adaptive patterns of the retina and the lens

**Figure 3:**
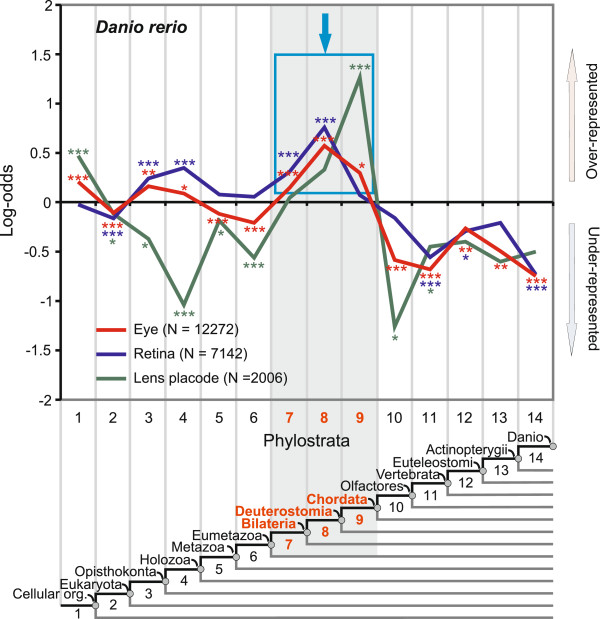
**Phylostratigraphic analysis of the zebrafish visual system.** A vertical grid depicts the 14 phylostrata that correspond to the phylogeny in the lower panel. In every phylostratum, the frequency of expression domains in an analyzed trait is compared to the frequency in the complete sample and deviations are shown by log-odds (y-axis). The total number of expression domains is given in parenthesis for each trait. The blue frame and the arrow denote dominant overrepresentation peaks. Log-odds of zero denote that the frequency of expressions domains in a phylostratum equals the expected frequency estimated from the total number of expressions. Deviations from the expected frequencies were tested by a two-tailed hypergeometric test corrected for multiple comparisons by FDR at 0.05 level (*P < 0.05; **P < 0.01; ***P < 0.001).

Some animal lineages have well-developed eyes that contain both the retina and the lens. The retina has a photoreceptive function, while the lens allows the formation of a clear image by focusing the light on the surface of the retina. In vertebrates these two parts have different developmental origins. The retina develops from the forebrain, while the lens is derived from the non-neural ectoderm of placodal origin [5,58]. Figure 3 portrays the phylostratigraphic profile for the complete visual system in zebrafish. The lens profile displays a dominant adaptive peak at the origin of chordates (ps9). Another smaller overrepresentation peak at the origin of all cellular organisms (ps1) implies some ancient pre-adaptations important for the lens (ps1). Interestingly, all of the known crystallins, which are important structural proteins that give rise to the optic properties of the lens, are located in ps1 and ps9 (Additional file 3: Table S2) [5]. It is also noticeable that adaptive signals for the lens are absent before and after the origin of chordates (Figure 3, ps2-8 and ps10-14).

For the zebrafish retina, the most prominent adaptive signal spreads over the Bilateria-Deuterostomia transition (ps7-8) (Figure 3). This result corroborates the idea that photoreceptive cells in vertebrate retinas are homologous to similar cells in other deuterostome and protostome lineages [1,2,5,59]. The two earlier signals are evident at the Opisthokonta-Holozoa transition (ps3-ps4), coinciding with the position of the basic retina regulatory network (Additional file 1: Figure S1) and with the evolutionary origin of phototaxis in the unicellular ancestors of animals [60]. Interestingly, the comparison of the dominant adaptive peaks in the lens and retina profiles suggests a directionality of the evolutionary change in the visual system, where the adaptive peak in the lens (ps9) follows those in the retina (ps7-8). Retina first and lens later is a common assumption in modeling of the evolution of eyes [61]. To further scrutinize the robustness of the recovered adaptive signals, we performed an analysis of the complete eye (Figure 3). All initially found signals were still present in this expanded dataset, indicating their importance for the evolution of vertebrate vision. Finally, we also note that disparate adaptive peaks of the lens and retina in vertebrates favor the idea that these structures have different evolutionary origins [17].

### A link between the sense of smell, hearing and balance and the origin of Olfactores

**Figure 4:**
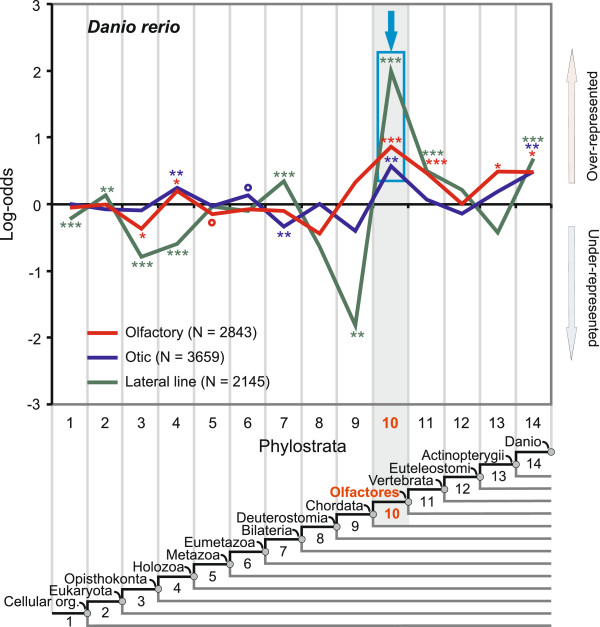
**Phylostratigraphic analysis of the zebrafish olfactory, otic and lateral line systems.** A vertical grid depicts the 14 phylostrata that correspond to the phylogeny in the lower panel. In every phylostratum, the frequency of expression domains in an analyzed trait is compared to the frequency in the complete sample and deviations are shown by log-odds (y-axis). The total number of expression domains is given in parenthesis for each trait. The blue frame and the arrow denote dominant overrepresentation peaks. Log-odds of zero denote that the frequency of expressions domains in a phylostratum equals the expected frequency estimated from the total number of expressions. Deviations from the expected frequencies were tested by a two-tailed hypergeometric test corrected for multiple comparisons by FDR at 0.05 level (*P < 0.05; **P < 0.01; ***P < 0.001, empty circles denotes significance before FDR correction at 0.05 level).

The functional role of the olfactory system is to sense volatile chemical compounds coming from the environment. It is thought that this system played an important role in the development of the vertebrate brain [62]. Figure 4 shows the recovered phylostratigraphic profile for the zebrafish olfactory system. Phylostratum 10, covering the last common ancestor of the Olfactores, harbors the strongest adaptive peak of the developing olfactory system (Figure 4). It is probably not a coincidence that the olfactory system peaks at this particular taxon, named precisely after the olfactory function [63]. This dominant peak in our analysis adds to the evidence that olfaction in tunicates and vertebrates is a synapomorphy [63]. A relatively weak earlier peak can be seen at the holozoan ancestor (ps4). This is suggestive, given that olfaction in choanoflagellates, a unicellular group (ps4) that is evolutionarily closest to animals, is thought to be linked to the origin of animal multicellularity [12,64,65]. Less prominent peaks, though still significant, are also apparent at the origin of vertebrates (ps11) as well as along the fish lineage (ps13-ps14) (Figure 4). Although most of the genes in these periods have unknown functions [56], we were able to recover six olfactory receptor genes (*ora1-6*) in ps11 and ps13 (Additional file 3: Table S2) that are relevant for pheromone signaling. These genes probably reflect an adaptive need for an upgrade of olfactory communications in the context of a more complex behavior within the vertebrate lineage [66].

The otic (vestibuloauditory) system is responsible for the sense of hearing and balance and its sensory ability is achieved by hair cells. Similar to the olfactory system, we recovered the strongest overrepresentation peak for the otic system at the origin of Olfactores (ps10) (Figure 4). Moreover, the overall profiles are similar between these two systems. For example, an early significant peak is present at the holozoan ancestor (ps4), and a late adaptive signal at the ancestor of zebrafish (ps14). This late signal (ps14) in the otic system correlates well with the origin of hearing specializations, such as Weberian ossicles, in some fish groups [67]. Although the otic system mainly develops from the otic placode, the neural crest contributes glial cells to the statoacustic ganglion (Figure 1). To see if the statoacustic ganglion has some specific pattern, we analyzed the statoacustic ganglion and the rest of the otic system separately (Additional file 4: Figure S2). While the purely placodal part has a phylostratigraphic profile indistinguishable from the total otic system, the statoacustic ganglion shows a special pattern, where the strongest peak is visible at the origin of vertebrates (ps11) (Additional file 4: Figure S2). This dominant adaptive signature at the origin of vertebrates (ps11) is a general characteristic of neural crest-derived tissues (see below).

In addition to the otic system, hair cells also perform a sensory function in the lateral line. This placode-derived sensory system is present only in aquatic vertebrates, and is responsible for the detection of movements and vibrations in the surrounding water [67]. Again, the profile for the lateral line is fairly similar to the olfactory and the otic system (Figure 4). The dominant peak is again at the origin of Olfactores (ps10), and later signals are visible at the origins of vertebrates (ps11) and zebrafish (ps14). Within ps14 we recovered the gene *phoenix* that was found to be involved in the regeneration of sensory hair cells in zebrafish. Interestingly, this feature is lacking in mammals, making them more prone to postnatal hearing disorders [68]. Relatively weak early signals are also present at ps2 and ps7.

Taken together, our phylostratigraphic profiles suggest similar evolutionary trajectories for olfactory, otic, and lateral line systems in zebrafish. The dominant signals of the olfactory, otic, and lateral line systems at the ancestor of Olfactores (ps10) agree well with some studies that suggest the presence of homologous structures in tunicates [3,28,30,69].

### A vertebrate-specific signature in the trigeminal system and the adenohypophysis

**Figure 5:**
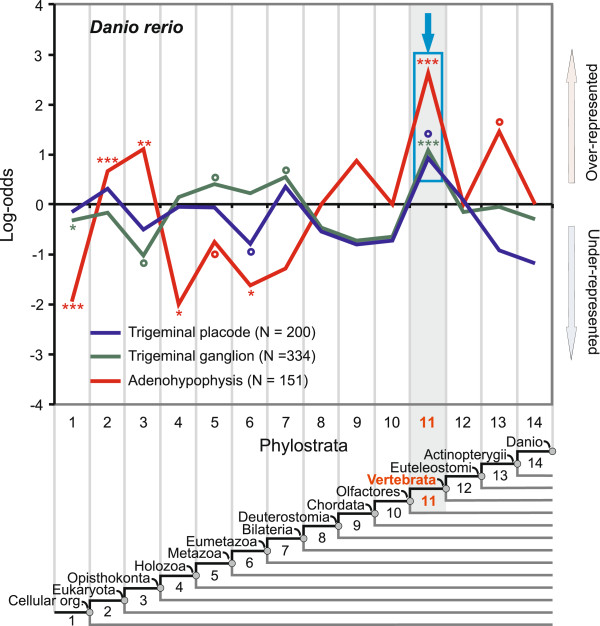
**Phylostratigraphic analysis of the zebrafish adenohypophysis and the trigeminal system.** A vertical grid depicts 14 phylostrata that correspond to the phylogeny in the lower panel. In every phylostratum, the frequency of expression domains in an analyzed trait is compared to the frequency in the complete sample and deviations are shown by log-odds (y-axis). The total number of expression domains is given for each trait in parenthesis. The blue frames and the arrows denote dominant overrepresentation peaks. Log-odds of zero denote that the frequency of expressions domains in a phylostratum equals the expected frequency estimated from the total number of expressions. Deviations from the expected frequencies were tested by a two-tailed hypergeometric test corrected for multiple comparisons by FDR at 0.05 level (*P < 0.05; **P < 0.01; ***P < 0.001, empty circles denotes significance before FDR correction at 0.05 level).

The trigeminal system innervates facial muscles and is responsible for sensations of touch, pain, and temperature in the frontal part of the vertebrate head. In zebrafish it is of mixed placodal and neural crest origin [19] (Figure 1). Figure 5 shows the strongest adaptive peak for both placodal and neural crest parts of the zebrafish trigeminal system at the vertebrate ancestor (ps11). This is in agreement with previous studies that note a lack of evidence for trigeminal system homologues outside the vertebrates [17,18]. Another system that is commonly thought of as a vertebrate innovation are epibranchial ganglia [17,18]. These ganglia innervate facial muscles and the pharynx and are responsible for the sense of taste. However, the scarcity of currently available expression data in the critical part of the phylogeny (ps8-ps14) precluded further analysis of this structure in this study.

The adenohypophysis (anterior pituitary) is a placodal component of the hypophysis, which is a major organ in the endocrine system. Although the adenohypophysis is not part of the sensory system *sensu stricto*, it is tightly linked to the development of the placodal sensory structures and hence we analyzed it here [17,18]. Figure 5 shows the strongest adaptive pattern for the zebrafish adenohypophysis in the ancestor of the vertebrates (ps11). Most of the genes found here code for adenohypophysis-specific hormones (Additional file 3: Table S2). This is in line with the classical hypothesis that the adenohypophysis originated in the vertebrate ancestor (ps11) [70]. Although a homologous relationship between the sensory Hatscheck’s pit in cephalochordates and the vertebrate adenohypophysis has been suggested [2,71], we did not find a significant overrepresentation signal at the phylostratum covering the origin of chordates (ps9). The adenohypophysis curve also has pre-adaptive signals at eukaryote (ps2) and opisthokont ancestors (ps3). These signals mainly correspond to the conserved genes that constitute the placode regulatory network (Figure 2 and Additional file 1: Figure S1). Examples of genes in ps3 are *Pitx1* and *Foxe3*, which are required for the induction of the anterior placodal domain, and *Pit1*, which is required for the induction of the adenohypophyseal placode (Additional file 3: Table S2 and Additional file 5: Table S3).

### The placodal system predates the neural crest

**Figure 6:**
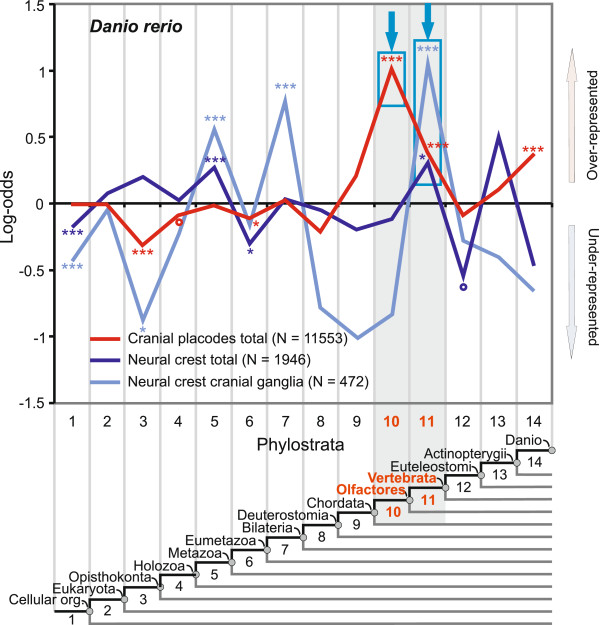
**A phylostratigraphic analysis of total placodal and neural crest tissues.** A vertical grid depicts 14 phylostrata that correspond to the phylogeny in the lower panel. In every phylostratum, the frequency of expression domains in an analyzed trait is compared to the frequency in the complete sample and deviations are shown by log-odds (y-axis). The total number of expression domains is given in parenthesis for each trait. The blue frames and the arrows denote dominant overrepresentation peaks. Log-odds of zero denote that the frequency of expressions domains in a phylostratum equals the expected frequency estimated from the total number of expressions. Deviations from the expected frequencies were tested by a two-tailed hypergeometric test corrected for multiple comparisons by FDR at 0.05 level (*P < 0.05; **P < 0.01; ***P < 0.001, empty circles denotes significance before FDR correction at 0.05 level).

Our analysis of the trigeminal and the statoacustic ganglion revealed a possibly unique macroevolutionary pattern for the neural crest (Figure 5 and Additional file 4: Figure S2). To explore this further, we analyzed sensory neural crest ganglia and all neural crest tissues. Figure 6 shows that the strongest significant adaptive peak for both the zebrafish neural crest cranial ganglia and the total neural crest is located at the ancestor of vertebrates (ps11). This result is in agreement with the traditional view that the neural crest originated in the vertebrate ancestor (ps11) [9,72]. The phylogeny before the advent of deuterostomes reveals early signals at the metazoan (ps5) and the bilaterian ancestors (ps7). These signals correlate in part with the position of the basic neural crest regulatory network that incorporates evolutionarily old genes (Figure 2 and Additional file 1: Figure S1).

**Figure 7:**
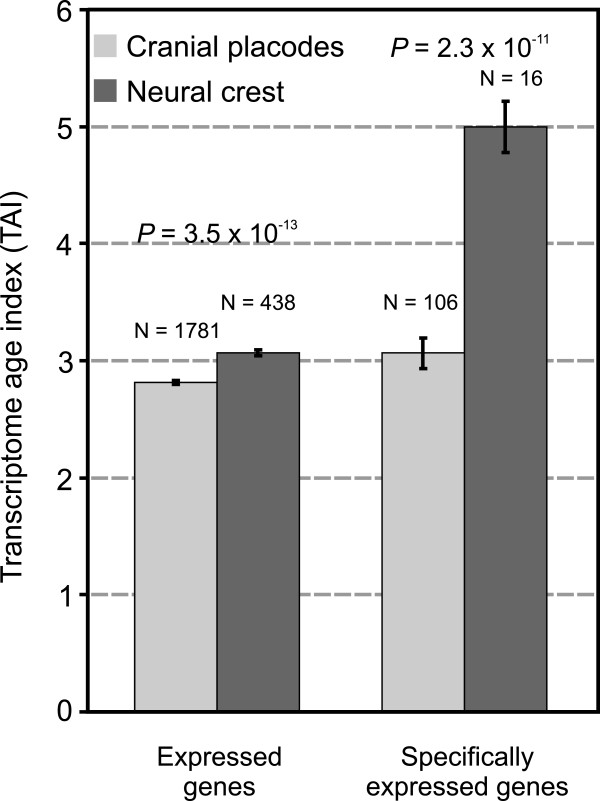
**Transcriptome age index analysis of the total placodal and neural crest tissues.** Phylogenetic age of the transcriptome measured by TAI is compared between the placodes and the neural crest. TAI values were calculated as previously described [43] using the zebrafish microarray expression data (see Methods). Note that a lower TAI value marks a phylogenetically older transcriptome. For every gene included in the analysis, microarray expression levels were averaged over ontogeny. Bars on the left compare all expressed genes in the placodes and the neural crest, whereas the comparison on the right is restricted to those genes with exclusive expression in these structures. The significance of differences was tested using the Student’s t-test. Error bars represent ±1 standard error of mean.

The evolutionary relationship between cranial placodes and the neural crest is an open question that has not been explicitly addressed so far. The phylogenetic position of the dominant peaks in the total analysis of the placodes and the neural crest suggests an independent origin of these tissues and their derivatives (Figure 6). If one assumes the most parsimonious scenario, the succession of the dominant overrepresentation peaks on the map supports the idea that the placodal system in total (ps10) evolved before the neural crest (ps11) (Figure 6). This is also evident in the analysis of individual placodes and neural crest derivatives (see above). However, it is also clear that the signal in the total neural crest, although significant, is much weaker compared to the sensory neural crest (Figure 6, ps11). To further test the robustness of the differences between the neural crest and the placodes we calculated the Transcriptome age index (TAI, Figure 7) [43,44,73]. This measure could also be used to test the directionality of evolutionary change by contrasting the phylogenetic age of the transcriptomes [73]. Initially, we included in the analysis only those genes that showed *in situ* hybridization expression pattern in the placodes (1781 gene) and the neural crest (438 genes) [57]. For every selected gene we estimated an expression level by averaging the microarray expression values across the zebrafish ontogeny [43]. Figure 7 shows that the cranial placodes express a phylogenetically older transcriptome compared to the neural crest (t-test, P = 3.5 x 10^-13^). However, as some of the compared genes are expressed in other tissues apart from the placodes and neural crest, we made a more stringent comparison. This refined analysis, where we included only the genes that are exclusively expressed in the placodes (106 genes) and the neural crest (16 genes), confirmed that the placodes express a phylogenetically older transcriptome (t-test, P = 2.3 x 10^-11^) (Figure 7). These results corroborate our phylostratigraphic analysis of the *in situ* expression data that suggest an independent origin of the placodes and the neural crest. Both analyses support the hypothesis that the placodal system emerged first, and that the neural crest was added later to the head sensory system (Figures 6 and 7).

## Discussion

**Figure 8:**
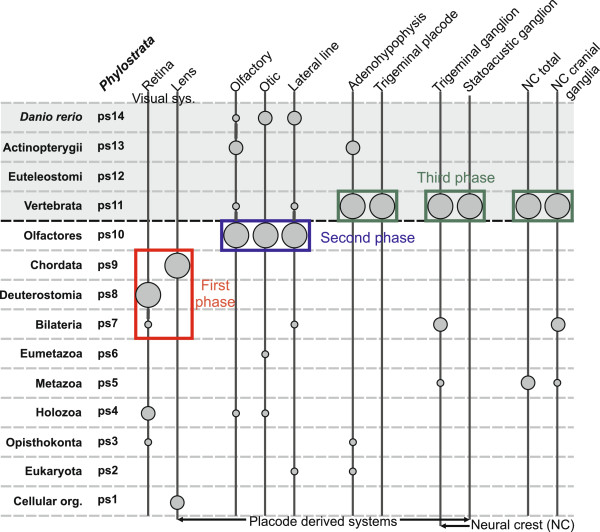
**Cranial placodes and sensory neural crest during head development in zebrafish.** Phylogenetic levels (phylostrata) from the ancestor of the cellular organisms (ps1) to the present day zebrafish (ps14) are shown at the left. The gray shaded area marks the vertebrate section of the phylogeny. For all studied parts of the sensory system (Figures 2, 3, 4, 5, 6 and 7) a simplified version of the phylostratigraphic profile is shown by the vertical lines and the corresponding circles. An adaptive signal with the strongest amplitude is represented by the largest circle, the second highest signal is marked by a medium size circle, and all other overrepresentation signals are marked by circles of the smallest size. Only statistically significant signals are shown. The three phases in the evolution of sensory system are labeled by different colors (first phase – red, second phase – blue, and third phase – green).

The relatively high resolution of our analysis allowed us to explore in great detail evolutionary trajectories of many distinct parts of the zebrafish head sensory system. To facilitate comparison across distinct phylostratigraphic profiles and to gain a global picture of the evolution of the sensory system in zebrafish, we plotted in a simplified way the statistically strongest adaptive signals of the individual analyses (Figure 8). This representation readily illustrates that many of the dominant adaptive signals in the analyzed head structures precede the evolutionary advent of vertebrates (Figure 8). Contrary to some traditional predictions [7,9,20], this result supports Butler’s Serial Transformation Hypothesis, which envisages a stepwise evolution of the vertebrate innovations and a pre-chordate origin of some head sensory structures [21,22]. Additionally, it is evident that the strongest adaptive signals could be grouped into three phases (Figure 8).

The first phase encompasses the transition from the bilaterian to the chordate ancestor (ps7-9). Here the visual system displays the strongest adaptive signals. This is, to our knowledge, the first genomic evidence that gives some credence to the idea that the visual system was the first complex sensory system to evolve in the lineage leading to the vertebrates [10,11,21]. However, the strong link between the present day zebrafish lens ontogeny and the origin of chordates (ps9) is somewhat puzzling if one looks at how lenses are distributed in the phylogeny among the extant deuterostomic lineages. First, a special type of calcitic lens is present in echinoderms (ps8) [5,74]. In contrast, lens structures are not described in cephalochordates (ps9) [71]. Then again, lens structures are present in both Olfactores lineages (ps10); i.e., vertebrates and tunicates, although these lens stuctures have most likely evolved convergently [75,76]. In this context, there are two scenarios that could explain the lens pattern with the strong adaptive peak at the origin of chordates (ps9). One possibility is that the chordate ancestor already possessed the lens. Under this hypothesis the absence of lenses in the modern cephalochordates could be a result of the secondary loss. This is not improbable given that lenses are known to evolve and also to get lost rapidly in different animal lineages [61], and that the body plan of cephalochordates has many derived features due to their semi-sessile way of life [5,71]. The alternative scenario assumes that lens related proteins evolved outside the placodal context and were only later recruited to the placodal tissue. For example, in ascidians there is an association between the lens proteins (crystallins) and the neural plate photoreceptors [75,76]. At present it is hard to discern which of these hypotheses is more likely. However, under the assumption that the evolutionary trajectories are to some extent mirrored in development, our phylostratigraphic patterns are consistent with the developmental finding that all placodes are initially specified as lens [32].

The early adaptive signals for the retina (Figure 8, ps7 and ps8) agree with the finding that some cell types in the vertebrate retina have deep roots within the bilaterian lineage [1,2,59]. In the context of the previous comparative evidence, which suggests that the vertebrate retina evolved from an unpaired frontal eye of the chordate ancestor [59], these signals most probably mark some important adaptations in the ancient photoreceptive system that are still retained in the retina of modern vertebrates.

The second prominent phase in the evolution of the sensory system corresponds to the origin of Olfactores (ps10, Figure 8). Here the olfactory, otic, and lateral line systems show dominant signals. This agrees well with studies that suggest a homology between the vertebrate placodes and the developing oral and atrial siphons in tunicates [3,14,28,30,69]. If this scenario is correct, it follows that the potential to build several placode derived sensory organs was already achieved at the origin of Olfactores. However, it is hard to say anything specific about the sensory complexity in the ancestor of Olfactores since it is not clear to what extent the sensory structures in modern tunicates are the result of a secondary reduction due to a sessile life style[3,24]. Finally, our results give support to the hypothesis that the otic and the lateral line placodes share a common evolutionary origin [77].

Another interesting property of the ps10 as well as the two earlier phylostrata (ps8-ps9) is related to their relatively small gene content (Figure 2). Although smaller sample sizes inherently bring higher noise levels, it is evident that the adaptive signals in these periods are strongly statistically supported. This resistance to the noise demonstrates a very strong footprint left by the adaptive events in ps8-10.

The origin of vertebrates (ps11) marks the final phase in the vertebrate sensory system evolution (Figure 8). Here dominant adaptive peaks are displayed by the two placodal tissues, trigeminal system and adenohypophysis, as well as the neural crest (Figure 8, ps11). Concerning the trigeminal system, this result supports previous work that argued for an entirely vertebrate character of the trigeminal system [17,18]. Hatscheck’s pit, a neurosecretory tissue located at the base of the brain in cephalochordates, has been proposed to be homologous to the adenohypophysis in vertebrates because it expresses regulatory genes crucial for the developmental induction of the adenohypophysis [2,71]. Contrary to this view, cephalochordate genome studies do not reveal homologues of the adenohypophyseal hormones, leading to the hypothesis that the functional adenohypophysis emerged within the vertebrates [72,78]. The dominant peak for adenohypophysis that we recovered at the ps11 supports the idea that a *bona fide* adenohypophysis emerged in vertebrates. However, these results do not necessarily preclude homology of the adenohypophyseal placode with Hatschek's pit at the level of the ectodermal regions that give rise to these structures. In total, the current evidence suggests that the trigeminal system, the sensory neural crest, and the adenohypophysis evolved at the base of the vertebrate lineage.

Our phylostratigraphic profile revealed an intimate link between the origin of the neural crest and the ancestor of vertebrates. This contrasts with some studies that report neural crest-like cells in tunicates [34,36]. However, methodological uncertainty, incompleteness of the neural crest gene regulatory network in tunicates, and, most importantly, the mesodermal origin of these neural crest-like cells call into question their homology to the vertebrate neural crest [15,38,79]. Taking this into account, a recent study looked for a different cell lineage that would represent a better candidate for a neural crest homolog in tunicates [38]. Developmental analysis of an ectodermal cell lineage at the neural plate border in *Ciona intestinalis* revealed that the targeted misexpression of *Twist* induces a migratory phenotype in this cell type. This finding indicates that the regulatory change of only one gene in tunicates could promote migratory properties of the cells at the neural plate border. However, it remains clear that in wild type tunicates these cells do not migrate, regardless of the seemingly short mutational distance required to reach migratory potential in the mutants. Furthermore, although migratory potential is a necessary property of the neural crest, it is not a defining feature of the neural crest phenotype [37]. For instance, the long distance migration of the neural crest cells in vertebrates is followed by differentiation into various cell types; a property not seen in the *Twist* reprogrammed tunicate cells [38]. All of this argues against a *bona fide* neural crest in tunicates. To understand vertebrate sensory system evolution it is also important to note that possible homologues of the neurogenic neural crest were never proposed outside vertebrates [13]. Taken together, we conclude that at present both developmental and phylostratigraphic evidence favors a scenario where the neural crest is a genuine vertebrate innovation.

A common evolutionary origin of the cranial placodes and the neural crest has been proposed on the basis of developmental similarities, which include migratory capacity, production of similar cell types, and their common emergence during early development at the borders of the neural plate [7]. However, these similarities could result from a convergent use of transcription factors. This would indicate an independent evolutionary origin of the neural crest and cranial placodes [39]. There are also some obvious differences in the development of the cranial placodes and the neural crest. For instance, placodes develop from the non-neural ectoderm exclusively in the head and are specified *after* gastrulation. In contrast, the neural crest develops from the neural ectoderm both in the head and the trunk and is specified *during* gastrulation, suggesting an independent origin of this embryonic tissue [39]. In this study, both the phylostratigraphic profiles and the evolutionary age of transcriptomes, measured by the transcriptome age index (TAI), indicate that the placodal system evolved before the sensory neural crest [39]. To our knowledge this is the first genomic evidence that supports the hypothesis of a separate evolutionary origin for these tissues [13].

The emerging picture on the evolution of the sensory system in vertebrates also has paleoecological implications. A rise in complexity of the sensory systems in Cambrian animals is thought to be driven by the rise in complexity of the environment at that time [12]. It is a matter of debate which sensory system first contributed to the arms race that took place in the Cambrian environment [11,12]. Some authors suggest that the olfactory system was first [12], whereas other authors envisage that visual information (and consequently the visual system) was the primary driver of the arms race [10,11,80]. Our results support the latter view by suggesting that the visual organs were the first to reach complexity comparable to extant groups [80,81]. It is obvious that our results are limited to the evolutionary trajectory of vertebrates, nonetheless, some recent fossil reports indicate that the visual system was the first sensory system to evolve in arthropods as well [80,81]. The early, concomitant, and independent evolution of complex eyes in unrelated groups is to be expected if visual information played a crucial role in the early Cambrian environment [80,81]. As the environment got more complex, one could expect that other elaborated sensory systems, such as olfactory and auditory, evolved to keep pace with competing groups. The second and third phase of the vertebrate sensory system evolution that we uncovered here match with this hypothetical scenario (Figure 8).

The theoretical underpinnings and assumptions of the phylostratigraphic approach have been discussed previously [40,42,56]. However, some points related to the functional role of novel genes need further attention here. Although phylostratigraphy is based on the assumption that the origin and evolution of at least some morphological novelties is linked to the origin of novel genes, it is nevertheless astonishing that all the analyzed structures in the sensory system show a phylostratigraphic signal, evidenced by the presence of the statistically significant overrepresentations. This is not self-explanatory, as both regulatory changes and mutations in existing proteins have been considered important factors in morphological evolutionary change [82-84]. If these types of changes were the only or prevailing driver of morphological evolution, we would expect to see insignificant fluctuations around expected values on the phylostratigraphic maps, i.e., absence of a phylostratigraphic signal. However, this is not the case. The abundance of phylostratigraphic signals in the zebrafish sensory system demonstrates that a morphological change, other than regulatory rewiring and protein sequence substitutions, is frequently accompanied by the recruitment of novel genes [56]. Ultimately, the availability of raw material for the recruitment of novel genes should not be a limiting factor, given that a large pool of *de novo* protein sequences are readily available in organisms at any time [45,56].

Nevertheless, several sorts of bias could affect phylostratigraphic inference. The quality of mapping of genes on the phylogenetic tree inevitably depends on the reference sequences that are placed on the internodes of the phylogeny. Having this in mind, we optimized phylogenetic resolution in a way to keep the numbers of presently available sequences as high as possible on each internode of the phylogeny (Additional file 6: Table S4). In the future, this type of concern will be of less importance given that genomes of various phylogenetic affinities are accumulating at an accelerating rate. Thanks to this expansion of available sequence data, an increasingly higher phylogenetic resolution will also become possible.

To interpret the signals on the maps in this study we took the most parsimonious scenario by assuming that adaptive signals directly correspond to the phylostrata where they appear. However, one cannot expect this assumption to be correct in all cases. For example, it is possible that entire modules or regulatory networks evolved within one morphological context and that later in evolution they have been recruited by new morphological innovations. In these situations adaptive signals would appear earlier on the phylogeny and it would be hard to discern on the map if they represent pre-adaptive or adaptive events.

Lastly, although the zebrafish *in situ* hybridization dataset is currently the best resource of expression patterns for phylostratigraphy in vertebrates, it is far from being complete [57]. This dataset is assembled from a multitude of small studies that are often biased towards older, functionally annotated, and human disease genes (Additional file 3: Table S2). This implies that many orphan genes are still awaiting testing [56]. Expression patterns for these genes will certainly help in getting a more complete picture on the evolution of organ systems.

## Conclusions

To summarize, we showed here that phylostratigraphy could simultaneously recover adaptive footprints and a temporal sequence of origin for multiple organ systems in vertebrates. We demonstrated that evolutionary information hidden in genomes and anatomically annotated expression patterns could be successfully extracted by the phylostratigraphic approach. At present, a limiting factor for this approach is the availability of anatomically annotated *in situ* hybridization patterns that cover a substantial part of the genome and the ontogeny. Future accessibility of high quality expression datasets of this type in different species will greatly increase possibilities for mining of macroevolutionary information and for evaluating the robustness of patterns reported here. Finally, our results show that evolution at the macroevolutionary scale involves periods that are both marked by bursts of morphological innovations and recruitment of novel genes.

## Methods

### Phylostratigraphic analyses

A full account of the phylostratigraphic procedure is described in previous work [40-45]. Here we retrieved the *Danio rerio* (zebrafish) protein sequences (20378 genes) from the ZFIN database [57]. The full set of analyzed genes is listed in Additional file 5: Table S3. We compared sequences of these proteins against the non-redundant (nr) database (NCBI) by the blastp algorithm at the e-value cutoff of 1e-03 [85]. This database represents the most exhaustive set of known proteins across all organisms and therefore is the most suitable dataset for phylostratigraphic analysis. Prior to performing sequence similarity searches, we removed from the database all sequences of viral or unknown taxonomic origin, as well as those from metazoan taxa with a currently unreliable phylogenetic position (Myxozoa, Mesozoa, Chaetognatha and Placozoa). Following this cleanup procedure, we filled up the nr database with sequenced genomes that were not present in the database but were otherwise publicly available. The final database contained 6,252,405 protein sequences. For the number of sequences on each node see Additional file 6: Table S4*.*

Using the obtained BLAST output we mapped the genes onto the consensus phylogeny. We used phylogenetically the most distant blast match above the significance threshold (blast e-value less than 1e-03) as the criteria to assign the evolutionary origin to a gene. This is a quite conservative method of sorting genes that aims to catch a novelty in the protein sequence space [40,42,56]. Our choice of internodes in the phylogeny is a result of balancing between the robustness of these internodes in the phylogenetic studies [46-51], the availability of the sequence data for the sequence similarity searches, and the importance of the evolutionary transitions (Figure 1). Our consensus phylogeny spans over 14 evolutionary levels (phylostrata) starting from the origin of cellular organisms (ps1) and ending at the origin of the zebrafish (ps14) (Additional file 5: Table S3). To further improve the quality of BLAST searches, we performed a TBLASTN screen (E-value cutoff 1e-03) against the available TRACE and EST archives at the internodes where the number of sequences was relatively low (ps8 and ps11, Additional file 6: Table S4).

### Expression data and statistics

Among vertebrates, the zebrafish *in situ* hybridization dataset has the best coverage in terms of the screened number of genes and the coverage of the ontogeny (Additional file 5: Table S3). For example, in comparison to the zebrafish, the frog dataset has 3 times less genes with spatially restricted expression [86]. Similarly, the mouse dataset is restricted to only one later embryonic stage where organogenesis is already complete [87]. As both frog and mouse datasets have substantially lower numbers of expression domains compared to zebrafish in the part of the ontogeny which is critical for analysis of the sensory system (ps7-ps14), we opted to analyze only the zebrafish dataset. We retrieved from the ZFIN database the whole mount *in situ* hybridization expression data for 5592 zebrafish genes which show tissue-specific expression during ontogeny (Additional file 3: Table S2) [57]. In total, this set of genes contributes to 141,257 expression domains that are distributed over specific anatomic parts and the stages of the ontogeny (Additional file 2: Table S1 and Additional file 5: Table S3). We divided this total set of expression domains into subsets that correspond to the specific developing organs using zebrafish anatomical ontology (Additional file 3: Tables S2 and Additional file 5: Table S3). For every analyzed trait, we performed overrepresentation analyses of the expression domains by dividing the observed frequency in a phylostratum with the expected frequency estimated from the total dataset [40-42]. Obtained deviations were depicted in the figures by log-odds ratios and their significance was tested by two-tailed hypergeometric tests [88], corrected for multiple comparisons via a false discovery rate (FDR) at the 0.05 level [89]. The details of the statistical analysis for the analyzed anatomical structures are available in the Additional file 3: Table S2. For the purpose of the cross-profile comparison between individual phylostratigraphic maps of the analyzed organs and tissues, we created a cumulative diagram where we depicted only significant overrepresentations (Figure 8). In this analysis, the adaptive signal that shows the highest peak in an individual profile is marked by the largest circle, the second highest peak is marked by a medium circle, and the remaining significant signals are shown as small circles.

### Transcriptome age index (TAI)

To compare the phylogenetic age of the transcriptome in the placodes and the neural crest, we calculated the Transcriptome age index (TAI) [43]. Within the framework of zebrafish phylogeny (14 phylostrata) TAI could take theoretically any value between 1 and 14. It should be noted that a TAI of 1 would mean that only genes from ps1 are present in the trascriptome. In contrast, a TAI of 14 would mean that only genes from ps14 are expressed. Therefore, lower TAI values correspond to a transcriptome where on average phylogenetically older genes are expressed. To calculate TAI we retrieved microarray expression data [43] only for genes that show *in situ* expression in the zebrafish placodes and the neural crest [57]. To further simplify the obtained dataset, we averaged microarray expression data over all ontogenetic stages [43]. This procedure yielded averaged microarray expression levels for 1781 placodal and 438 neural crest genes (Figure 7). To calculate the TAI we inserted these averaged expression levels together with the phylogenetic ranks of the corresponding genes into the TAI equation described previously [43]. We also generated a more stringent dataset by taking only genes that are exclusively expressed in the cranial placodes (106 genes) and the neural crest (16 genes). We estimated the significance of the TAI differences between the placodes and the neural crest using the Student’s t-test at the 0.05 level. Error bars represent ± one standard error of mean.

## Competing interests

The authors declare that they have no competing interests.

## Authors' contributions

MSŠ carried out bioinformatic analyses. VB helped to analyze the expression data. RB and VD contributed new analytic tools and performed the statistical analysis. TDL initiated, designed and coordinated the study. MSŠ, VB and TDL wrote the manuscript. All authors read and approved this manuscript.

## Supplementary Material

Additional file 1: Figure S1 Phylostratigraphic analysis of the key developmental genes involved in the development of placodes, neural crest and retina. A vertical grid depicts 14 phylostrata that correspond to the phylogeny in the lower panel. In every phylostratum, the frequency of genes in an analyzed trait is compared to the frequency in the complete genome and deviations are shown by log-odds (y-axis). The total number of genes is given in parenthesis for each trait. The blue frame and the arrow denote dominant overrepresentation peaks. Log-odds of zero denote that the frequency of conserved genes in a phylostratum equals the expected frequency estimated from the total number of genes. Deviations from the expected frequencies were tested by a two-tailed hypergeometric test corrected for multiple comparisons by FDR at 0.05 level (*P < 0.05; **P < 0.01; ***P < 0.001, empty circles denotes significance before FDR correction at 0.05 level). The actual numbers of key developmental genes that are taken from several studies [17,52,90] are in the table at the top. Click here for file

Additional file 2: Table S1 Phylogenetic summary of the zebrafish phylostratigraphic and expression data. Click here for file

Additional file 3:Table S2 Zebrafish expression dataset. Expression and anatomical data for zebrafish genes as well as statistical details in an xlsx file. Click here for file

Additional file 4: Figure S2 Phylostratigraphic analysis of the vestibuloauditory system. A vertical grid depicts 14 phylostrata that correspond to the phylogeny in the lower panel. In every phylostratum, the frequency of expression domains in an analyzed trait is compared to the frequency in the complete sample and deviations are shown by log-odds (y-axis). The total number of expression domains is given in parenthesis for each trait. The blue frames and arrows denote dominant overrepresentation peaks. Log-odds of zero denote that the frequency of expressions domains in a phylostratum equals the expected frequency estimated from the total number of expressions. Deviations from the expected frequencies were tested by a two-tailed hypergeometric test corrected for multiple comparisons by FDR at 0.05 level (*P < 0.05; **P < 0.01; ***P < 0.001, empty circles denotes significance before FDR correction at 0.05 level). Click here for file

Additional file 5: Table S3 Zebrafish phylostratigraphic dataset. The full list of the zebrafish genes and their phylogenetic rankings in an xls file. Click here for file

Additional file 6: Table S4 Phylogenetic summary of the database used in the sequence similarity searches of the zebrafish genes. Click here for file
