# Supplementary material for: Phylostratigraphic profiles reveal a deep evolutionary history of the vertebrate head sensory systems
Source: Front Zool. 2013 Apr 12;10:18. doi: 10.1186/1742-9994-10-18 (PMC3636138; doi:10.1186/1742-9994-10-18)
Supplement: Additional file 1: Figure S1 — Phylostratigraphic analysis of the key developmental genes involved in the development of placodes, neural crest and retina. A vertical grid depicts 14 phylostrata that correspond to the phylogeny in the lower panel. In every phylostratum, the frequency of genes in an analyzed trait is compared to the frequency in the complete genome and deviations are shown by log-odds (y-axis). The total number of genes is given in parenthesis for each trait. The blue frame and the arrow denote dominant overrepresentation peaks. Log-odds of zero denote that the frequency of conserved genes in a phylostratum equals the expected frequency estimated from the total number of genes. Deviations from the expected frequencies were tested by a two-tailed hypergeometric test corrected for multiple comparisons by FDR at 0.05 level (*P < 0.05; **P < 0.01; ***P < 0.001, empty circles denotes significance before FDR correction at 0.05 level). The actual numbers of key developmental genes that are taken from several studies [17,52,90] are in the table at the top. [file 1742-9994-10-18-S1.pdf]

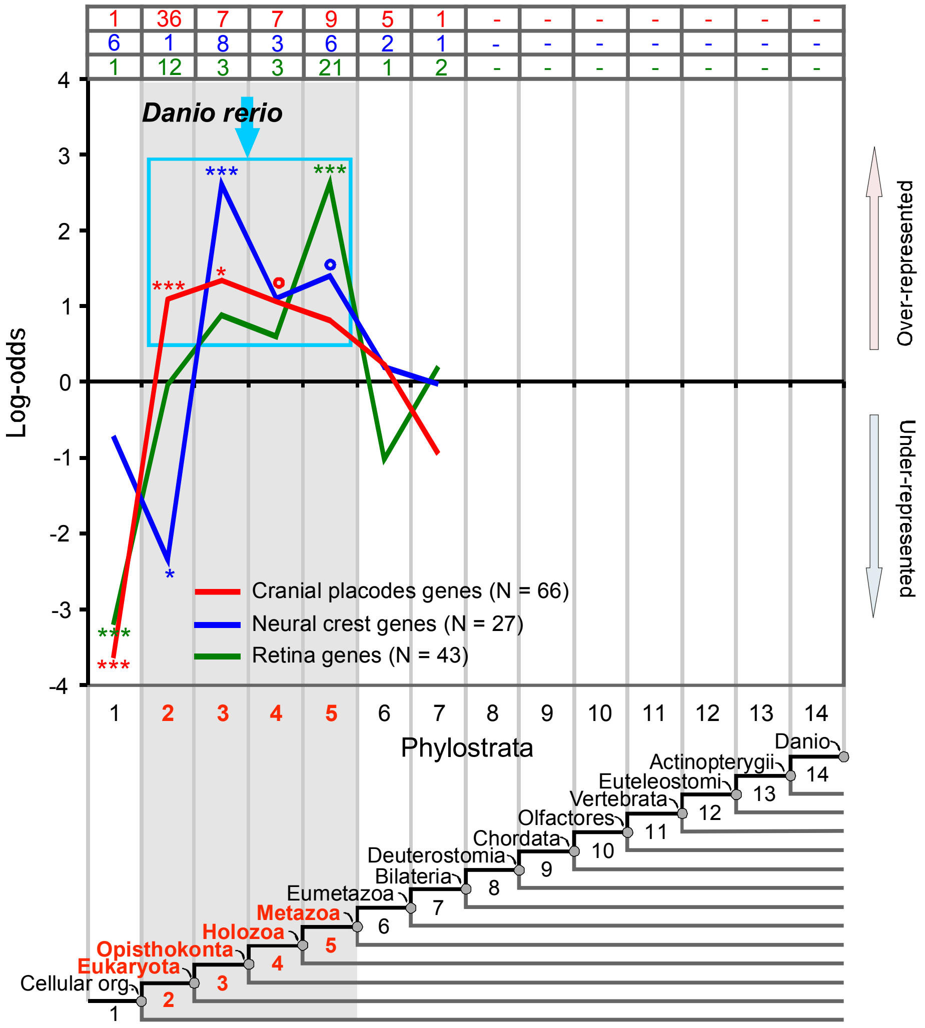

Figure S1 – Phylostratigraphic analysis of the key developmental genes involved in the development of placodes, neural crest and retina.
