# Supplementary material for: Phylostratigraphic profiles reveal a deep evolutionary history of the vertebrate head sensory systems
Source: Front Zool. 2013 Apr 12;10:18. doi: 10.1186/1742-9994-10-18 (PMC3636138; doi:10.1186/1742-9994-10-18)
Supplement: Additional file 2: Table S1 — Phylogenetic summary of the zebrafish phylostratigraphic and expression data. [file 1742-9994-10-18-S2.doc]

Table S1. Zebrafish phylostratigraphic and expression data.

| ***Danio rerio*** | | | | | | |
| --- | --- | --- | --- | --- | --- | --- |
| **Phylostrata** | | **Complete genome** | **Restricted expression** | | | |
| **Number** | **Internodes** | **Genes (%)** | **Genes (total) (%)** | **Expression domains (total)**  50497  38422  4633  6694  15032 | **Genes (pl + nc*) (%)** | **Expression domains (pl + nc*)** |
| **1** | Life before LCA of Cellular organisms - Cellular organisms | 7547 (37.0) | 2128 (38.1) | 50497 | 810 (36.5) | 50497 |
| **2** | Cellular organisms - Eukaryota | 5864 (28.8) | 1426 (25.5) | 38422 | 587 (26.4) | 38422 |
| **3** | Eukaryota - Opisthokonta | 622 (3.1) | 174 (3.1) | 4633 | 65 (2.9) | 4633 |
| **4** | Opisthokonta - Holozoa | 813 (4.0) | 256 (4.6) | 6694 | 104 (4.7) | 6694 |
| **5** | Holozoa - Metazoa | 1348 (6.6) | 502 (9.0) | 15032 | 210 (9.5) | 15032 |
| **6** | Metazoa - Eumetazoa | 1259 (6.2) | 346 (6.2) | 9061 | 145 (6.5) | 9061 |
| **7** | Eumetazoa - Bilateria | 779 (3.8) | 260 (4.6) | 6622 | 104 (4.7) | 6622 |
| **8** | Bilateria - Deuterostomia | 130 (0.4) | 25 (0.4) | 611 | 11 (0.5) | 611 |
| **9** | Deuterostomia - Chordata | 158 (0.8) | 39 (0.7) | 794 | 19 (0.9) | 794 |
| **10** | Chordata - Olfactores | 66 (0.3) | 29 (0.5) | 733 | 16 (0.7) | 733 |
| **11** | Olfactores - Vertebrata | 730 (3.6) | 168 (3.0) | 4135 | 72 (3.2) | 4135 |
| **12** | Vertebrata  -  Euteleostomi | 368 (1.8) | 118 (2.1) | 1978 | 38 (1.7) | 1978 |
| **13** | Euteleostomi  -  Actinopterygii | 216 (1.1) | 54 (1.0) | 893 | 21 (0.9) | 893 |
| **14** | Actinopterygii  -  Danio rerio | 478 (2.3) | 67 (1.2) | 1152 | 20 (0.9) | 1152 |
|  | **Total** | **20378 (100)** | **5592 (100)** | **141257** | **2222 (100)** | **13018** |

*pl + nc = cranial placodes and neural crest
