# Supplementary figures and images for: Phylostratigraphic profiles reveal a deep evolutionary history of the vertebrate head sensory systems
Source: Front Zool. 2013 Apr 12;10:18. doi: 10.1186/1742-9994-10-18 (PMC3636138; doi:10.1186/1742-9994-10-18)

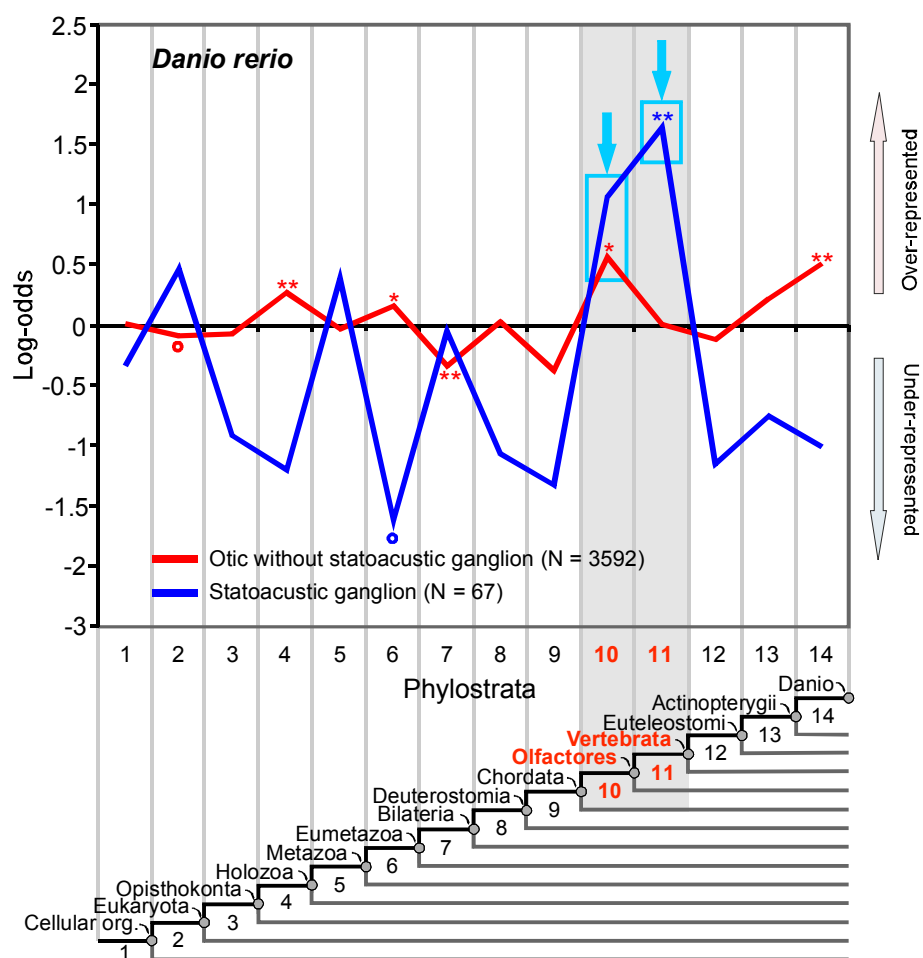

Figure S2 – Phylostratigraphic analysis of the vestibuloauditory system.

Supplement: Additional file 4: Figure S2 — Phylostratigraphic analysis of the vestibuloauditory system. A vertical grid depicts 14 phylostrata that correspond to the phylogeny in the lower panel. In every phylostratum, the frequency of expression domains in an analyzed trait is compared to the frequency in the complete sample and deviations are shown by log-odds (y-axis). The total number of expression domains is given in parenthesis for each trait. The blue frames and arrows denote dominant overrepresentation peaks. Log-odds of zero denote that the frequency of expressions domains in a phylostratum equals the expected frequency estimated from the total number of expressions. Deviations from the expected frequencies were tested by a two-tailed hypergeometric test corrected for multiple comparisons by FDR at 0.05 level (*P < 0.05; **P < 0.01; ***P < 0.001, empty circles denotes significance before FDR correction at 0.05 level). [file 1742-9994-10-18-S4.pdf]
