# Supplementary material for: Phylostratigraphic profiles reveal a deep evolutionary history of the vertebrate head sensory systems
Source: Front Zool. 2013 Apr 12;10:18. doi: 10.1186/1742-9994-10-18 (PMC3636138; doi:10.1186/1742-9994-10-18)
Supplement: Additional file 6: Table S4 — Phylogenetic summary of the database used in the sequence similarity searches of the zebrafish genes. [file 1742-9994-10-18-S6.doc]

Table S4. Contents of the database used in the sequence similarity searches of the zebrafish genes.

| **Phylostrata** | **Node** | **NCBI nr database sequences** | **Genomes included in nr database** |
| --- | --- | --- | --- |
| 14 | *Danio rerio* | 104716 | *Danio rerio* |
| 13 | Actinopterygii | 141574 | *Gasterosteus aculeatus, Oryzias latipes, Takifugu rubripes, Tetraodon nigroviridis* |
| 12 | Euteleostomi | 992219 | *27 mammalian and bird species* |
| 11 | Vertebrata | 4175 | Cyclostomata and Chondrichthyes (1967268 ESTs) |
| 10 | Olfactores | 206140 | *Ciona savignyi, Ciona intestinalis, Oikopleura dioica* |
| 9 | Chordata | 51742 | *Branchiostoma floridae* |
| 8 | Deuterostomia | 41135 | *Strongylocentrotus purpuratus, Saccoglossus kowalevskii* (202190 ESTs) |
| 7 | Bilateria | 575446 | *26 protostomic animals* |
| 6 | Eumetazoa | 146527 | *Aurelia aurita, Hydra magnipapillata, Hydra oligactis, Hydra viridissima, Nematostella vectensis* |
| 5 | Metazoa | 61533 | *Amphimedon queenslandica* |
| 4 | Holozoa | 29264 | *Monosiga brevicollis, Capsaspora owczarzaki, Salpingoeca rosetta* |
| 3 | Opisthokonta | 510446 | *46 fungal species* |
| 2 | Eukaryota | 1256015 | *33 plant and other eukaryotic species* |
| 1 | Cellular org. | 2131473 | ~ 27 archeal genomes  ~337 bacterial genomes |
|  | **Total** | **6252405** |  |
